# Supplementary material for: Spatially resolved transcriptomic profiling of degraded and challenging fresh frozen samples
Source: Nat Commun. 2023 Jan 31;14:509. doi: 10.1038/s41467-023-36071-5 (PMC9889806; doi:10.1038/s41467-023-36071-5)
Supplement: Supplementary file 1 — Supplementary Information [file 41467_2023_36071_MOESM1_ESM.pdf]

Supplementary

Supplementary Table 1: Samples used in this study with their corresponding ID, RNA Integrity Number (RIN) and DV200.

Sample Type	Sample ID	RIN	DV200 (%)
Mouse brain	MB	8.8	93
Prostate cancer	PC	10	100
Human lung	LNG1	7.1	90
Human lung	LNG2	6.8	89
Human colon	CLN1	5.1	77
Human colon	CLN2	4.5	77
Human small intestine	SI	7.8	86
Pediatric brain tumor	WNT medulloblastoma (PBT1)	7.1	90
Pediatric brain tumor	NOS (PBT2)	7	88
Mouse bone	P4	6.2	87
Mouse bone	P11	5.6	82

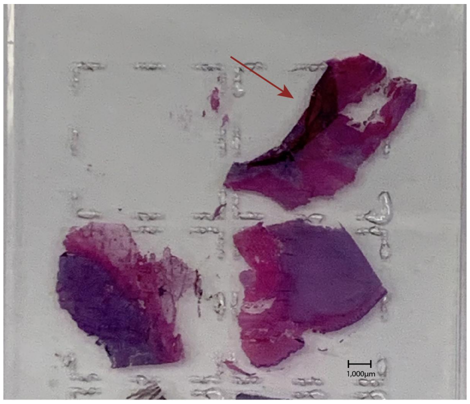

Supplementary Figure 1: Tissue detachment. Tissue section detachment from a spatial gene expression array indicated by an arrow right after staining steps. **(plus Supplementary Video 1)**.

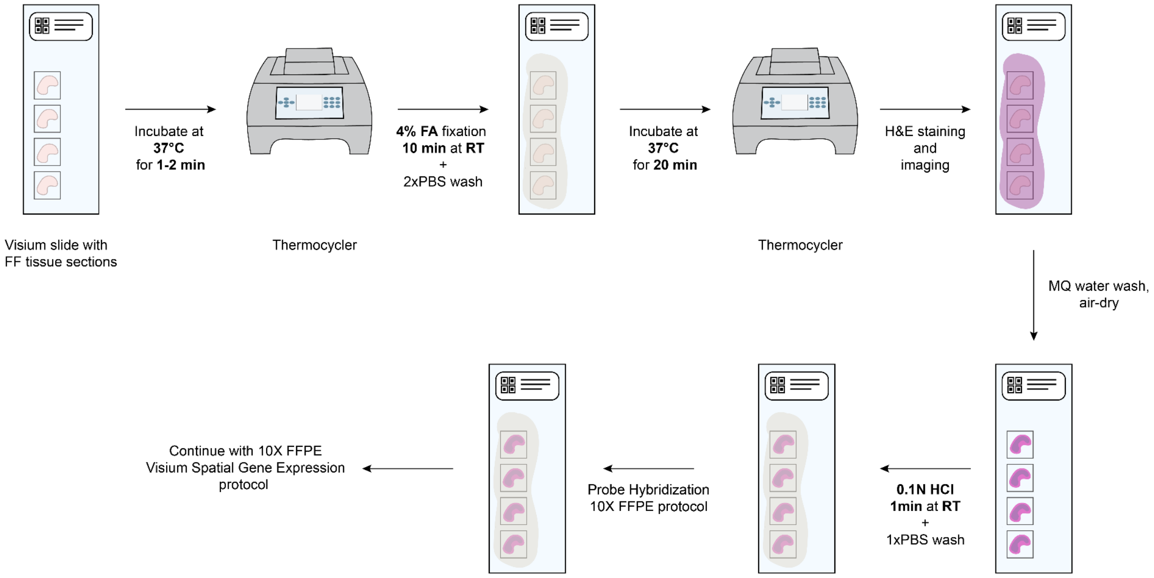

Supplementary Figure 2: Schematic overview of the RRST protocol workflow. FA refers to Formaldehyde, RT refers to room temperature.

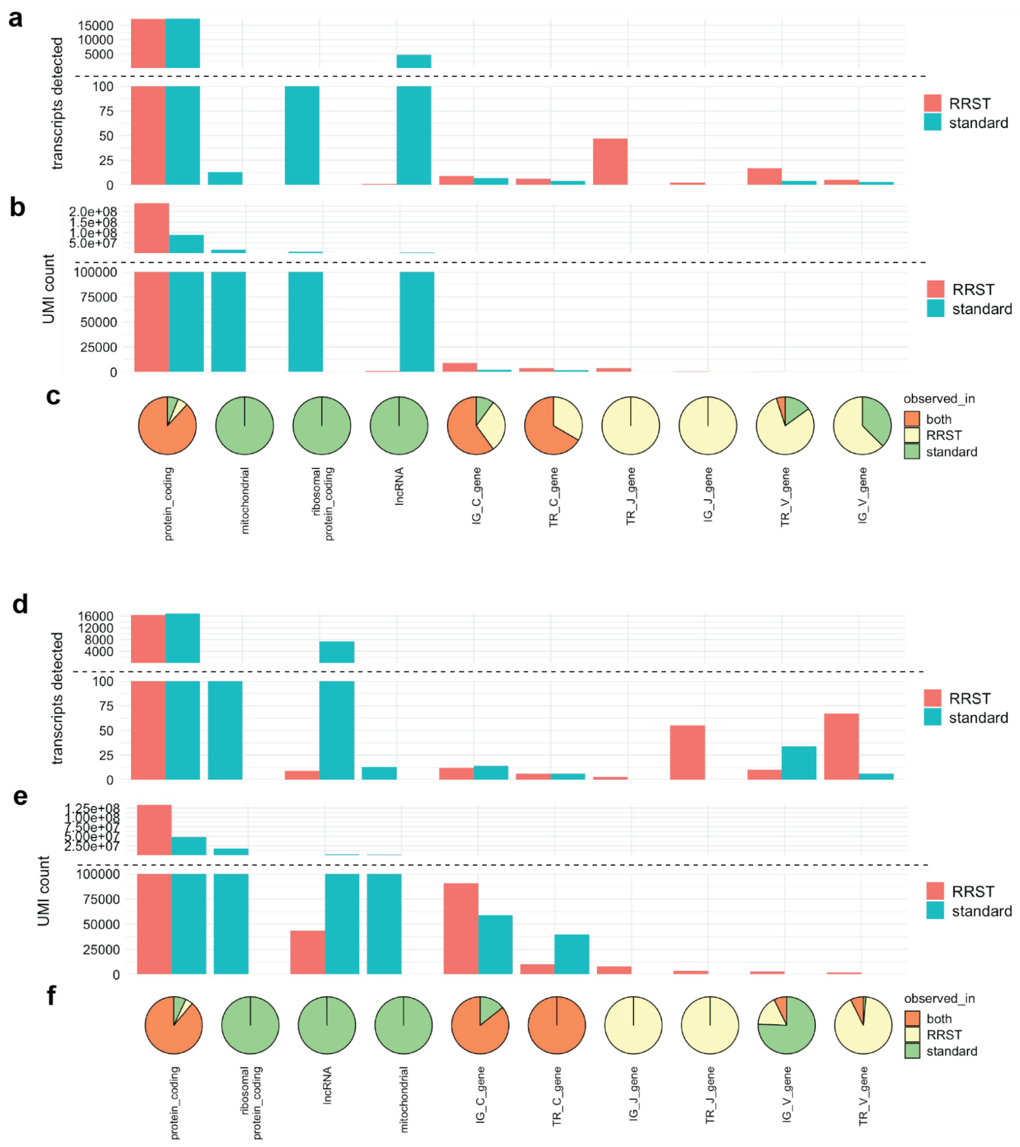

Supplementary Figure 3: Biotype of transcripts present in Visium and RRST mouse brain and prostate cancer datasets. a, b) Transcripts detected (**a**) and UMI counts (**b**) for each biotype in the mouse brain data (RRST n=4, standard n=4). The fill color of the bars corresponds to the protocol used. The y-axis has been cut to display bio types with fewer detected transcripts or UMI counts. **c)** Pie charts below each bar in **b** display the proportions of transcripts that are detected with both methods, RRST only or standard Visium only. For example, lncRNA, mitochondrial transcripts and ribosomal protein coding gene transcripts are only detected with the standard Visium protocol. **d, e)** Transcripts detected (**d**) and UMI counts (**e**) for each biotype in the prostate cancer data (RRST n=2, standard n=2). The fill color of the bars corresponds to the protocol used. The y-axis has been cut to display bio types with fewer detected transcripts or UMI counts. **f)** Pie charts below each bar in **e** display the proportions of transcripts that are detected with both methods, RRST only or standard Visium only.

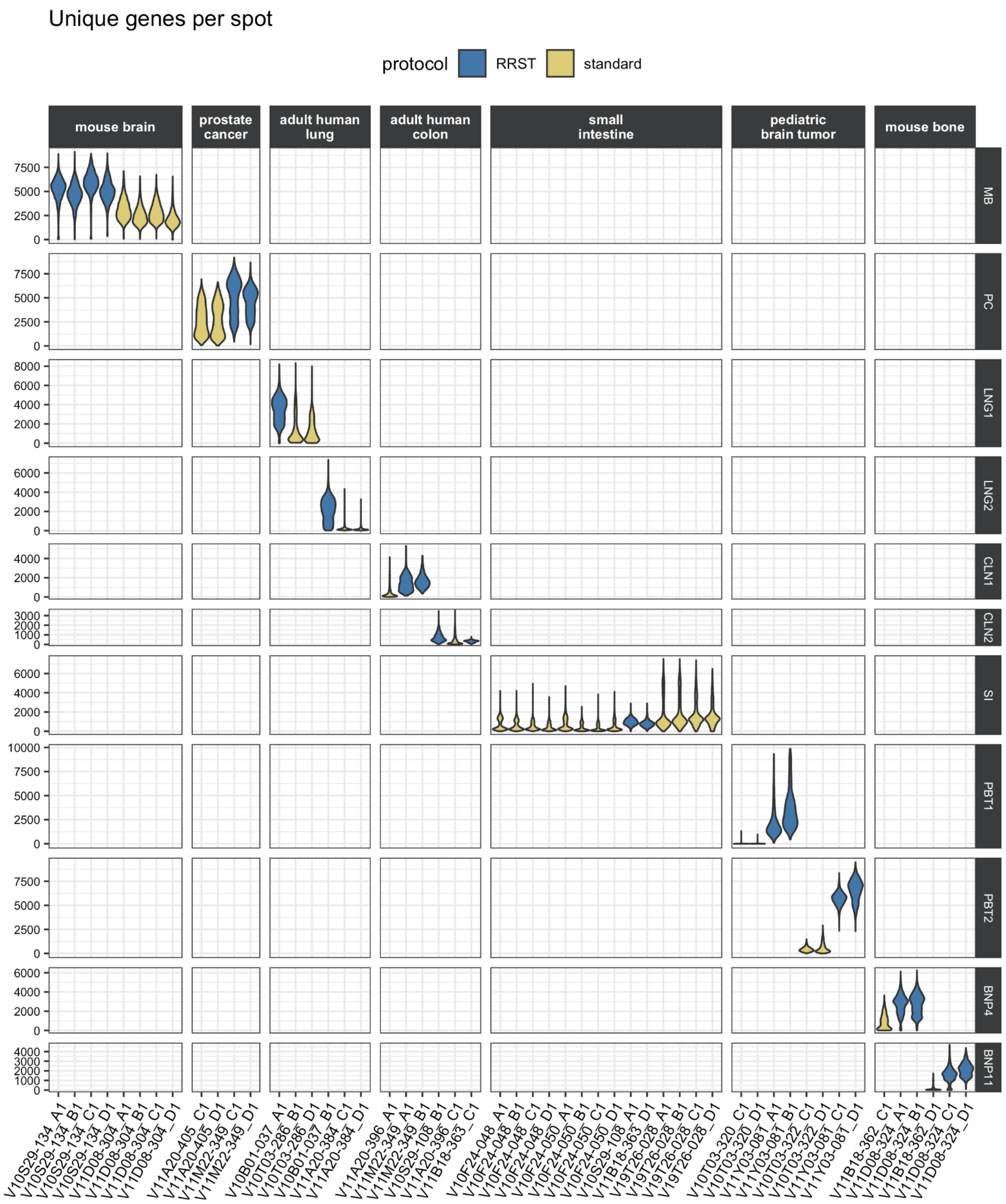

Supplementary Figure 4: Distribution of unique genes per spot for all SRT datasets included in this study visualized as violin plots. The fill color of the violin plots indicates the protocol used to generate the data. Columns are sorted by sample origin and rows are sorted by sample ID. MB, mouse brain; PC, prostate cancer; LNG, adult human lung; CLN, adult human colon; SI, adult human small intestine; PBT, pediatric brain tumor; BN, mouse bone.

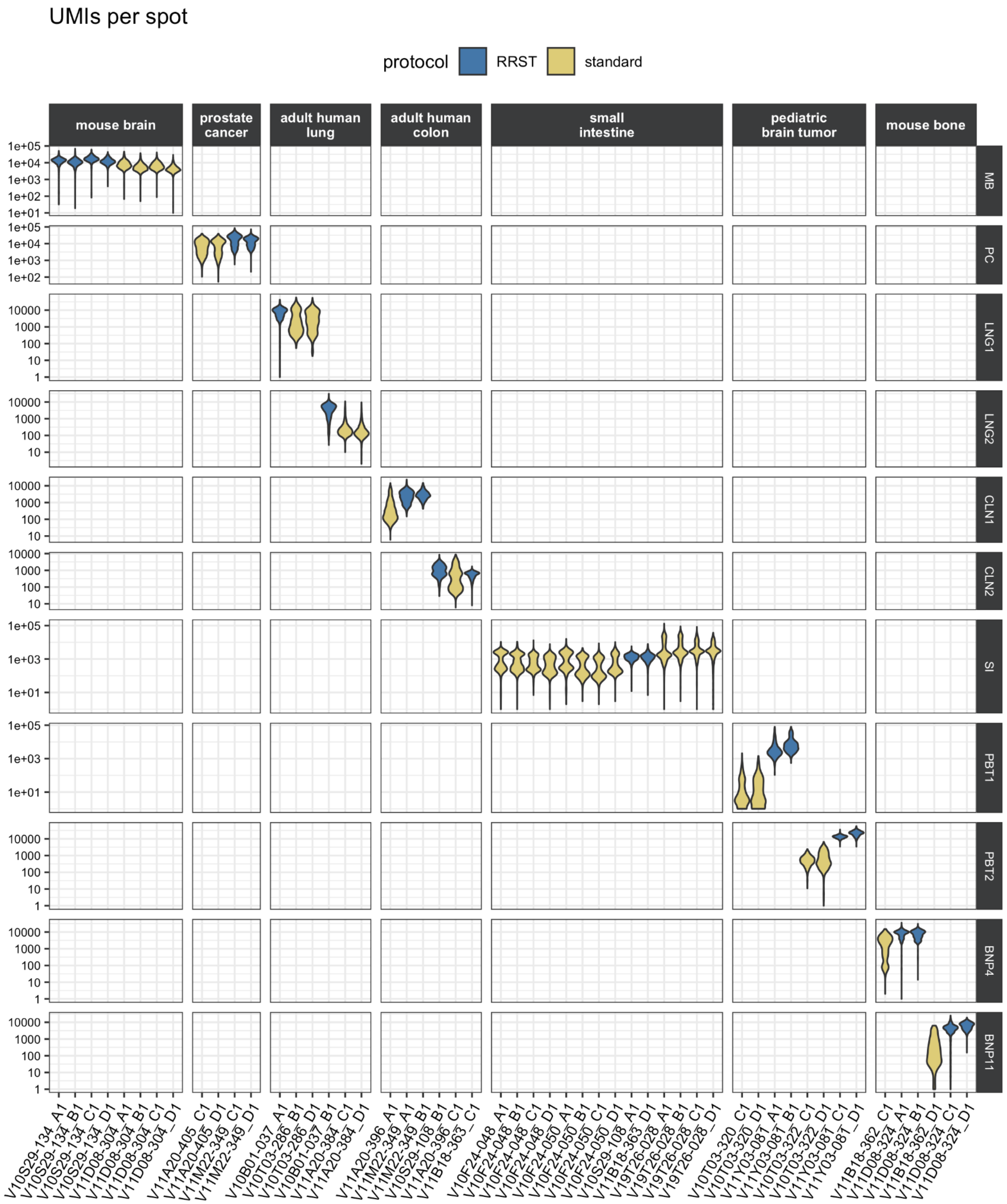

Supplementary Fig.5: Distribution of UMIs per spot for all SRT datasets included in this study visualized as violin plots. The fill color of the violin plots indicates the protocol used to generate the data. Columns are sorted by sample origin and rows are sorted by sample ID. MB, mouse brain; PC, prostate cancer; LNG, adult human lung; CLN, adult human colon; SI, adult human small intestine; PBT, pediatric brain tumor; BN, mouse bone.

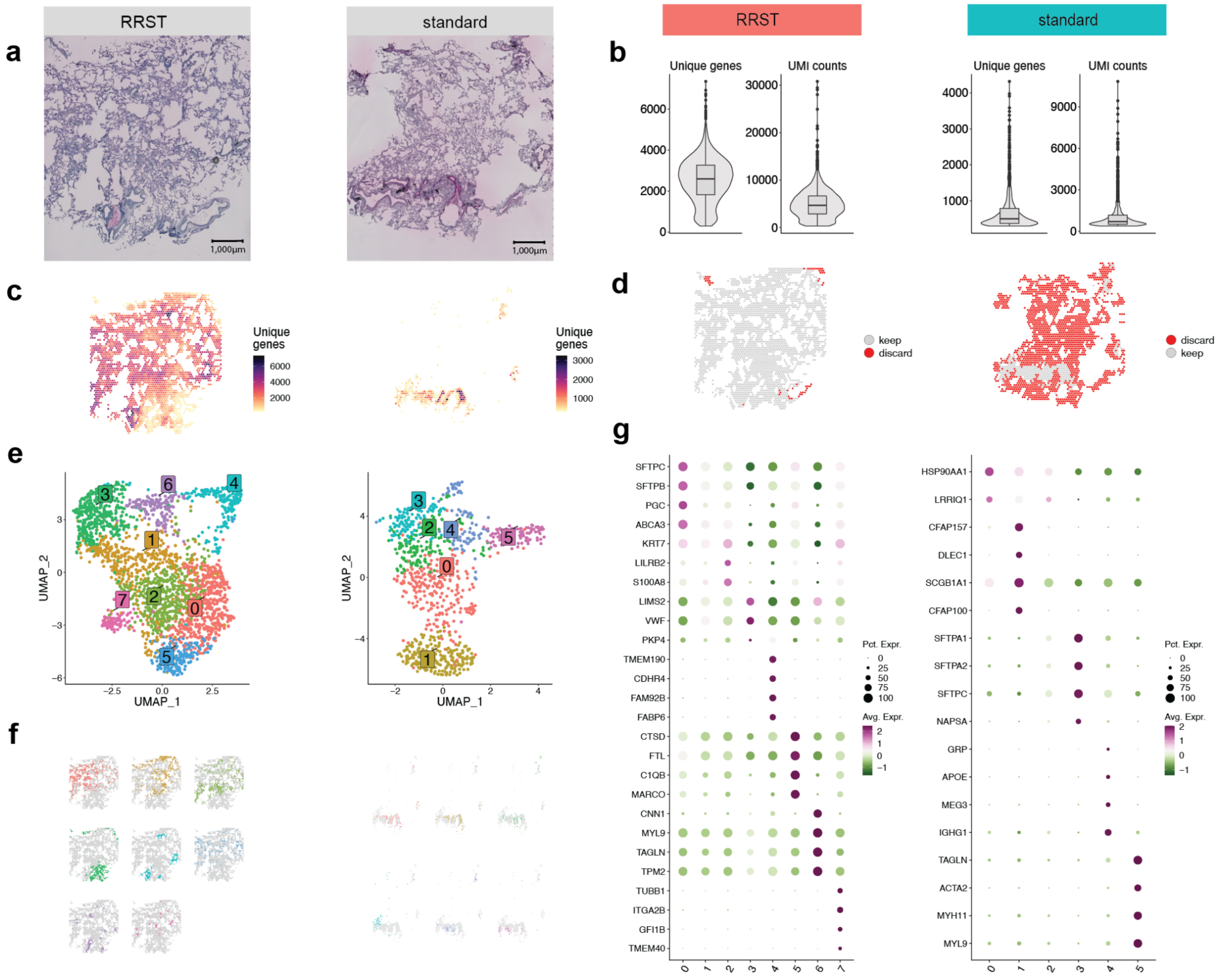

Supplementary Figure 6: Comparison of RRST and standard Visium in human adult lung tissue (LNG2). Each subplot shows the RRST data on the left side and the standard Visium data on the right side. **a)** H&E images of two representative tissue sections from the same tissue block. **b)** Violin/box plots showing the distribution of unique genes and UMI counts for RRST (n=1) and standard Visium (n=2) data generated from consecutive tissue sections from the same lung tissue specimen. The y-axis is shown in log10 scale. Box plots are presented as median values where the lower and upper bounds are the 25th and 75th percentiles. The upper and lower limits of the boxplots are defined by the closest value no further than 1.5*IQR (inter-quartile range) from the closest bound. Values outside of the upper and lower limits are highlighted as outliers. **c)** Unique genes per spot mapped on tissue coordinates. **d)** Spatial visualization showing what spots were discarded due to low quality (less than 300 unique genes detected). **e)** UMAP embedding of adult lung data colored by clusters detected by unsupervised graph-based clustering (louvain). **f)** Split view of clusters (same as in **e**) mapped on tissue coordinates. **g)** Dot plots of the top marker genes for each cluster.

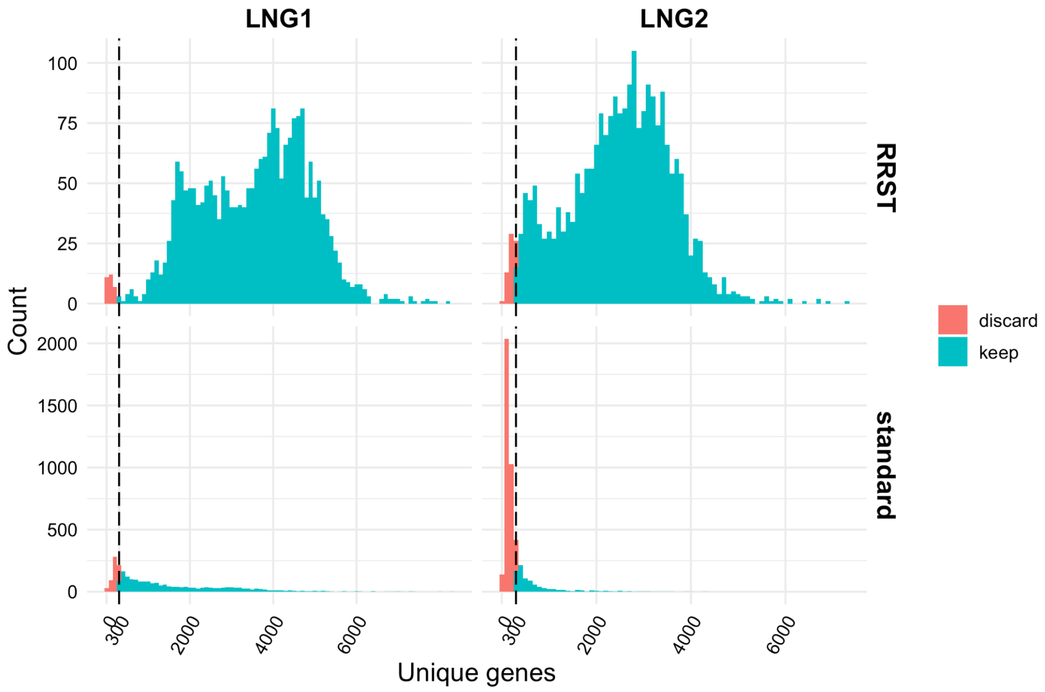

Supplementary Figure 7: Histograms showing the distribution of unique genes per spot in lung tissue data obtained with RRST and standard Visium. A cutoff threshold of 300 was used as a filtering threshold to remove low quality spots. Blue bars represent spots that passed the cutoff threshold whereas red bars represent spots that were discarded. ~1.3% - 2.3% of spots were discarded from the two RRST datasets and ~21%-81% of spots were discarded for the standard Visium dataset.

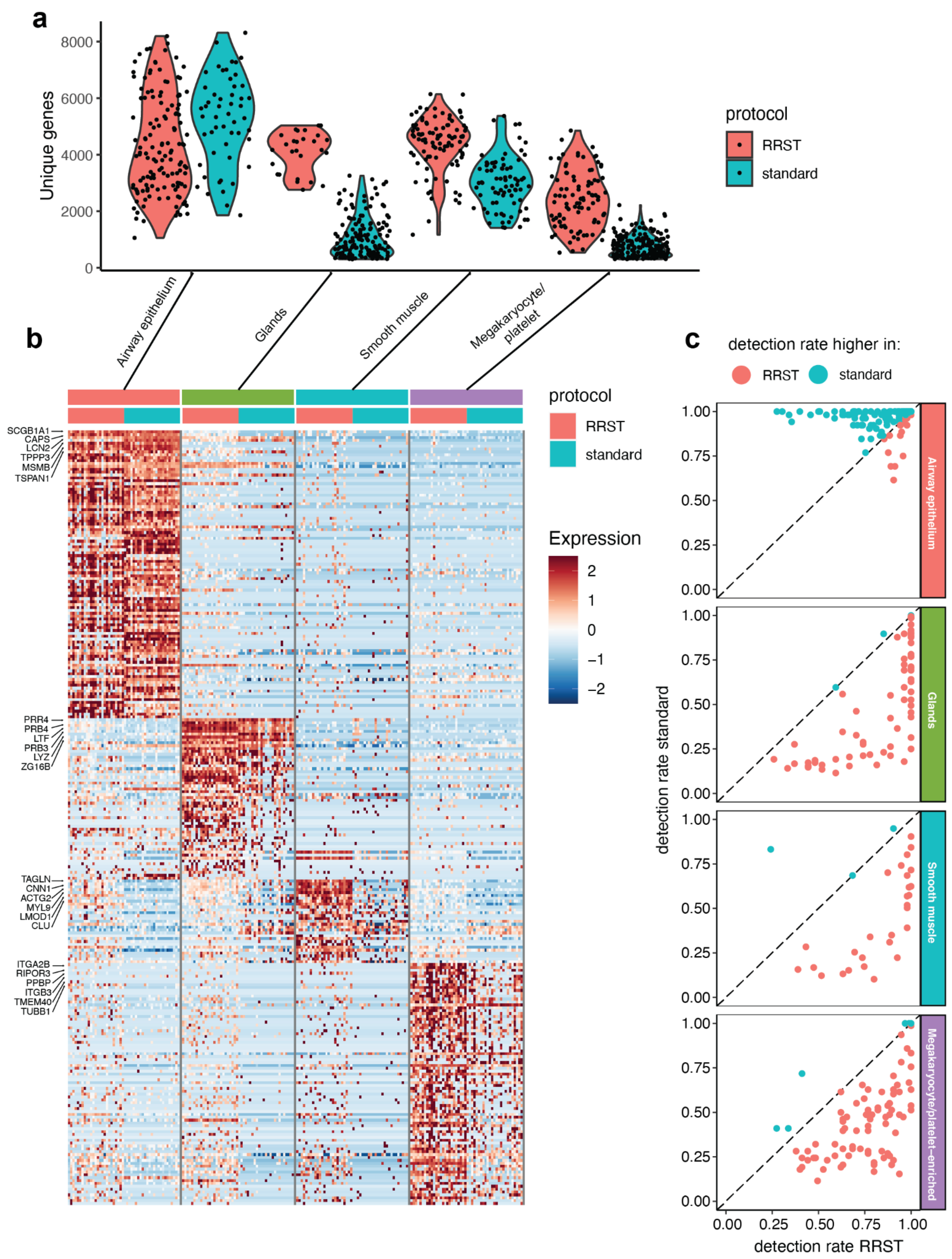

Supplementary Figure 8: Visualization of cluster marker genes identified in adult human lung data (LNG1). a) Violin plots showing the distribution of unique genes per spot within each cluster and condition. **b)** Heatmap showing the top differentially expressed genes (DEGs) selected by average log fold-changes across four annotated clusters. The color above the heat map specifies the cluster annotations and the protocol for the spots. The maximum number of selected DEGs per cluster is 100. The top 6 marker genes per cluster are highlighted next to the heat map. **c)** Gene-gene scatter plot showing detection rates for the DEGs (same as in **a**) within each cluster. Genes are colored based on whether their detection rates are higher in one of the two protocols.

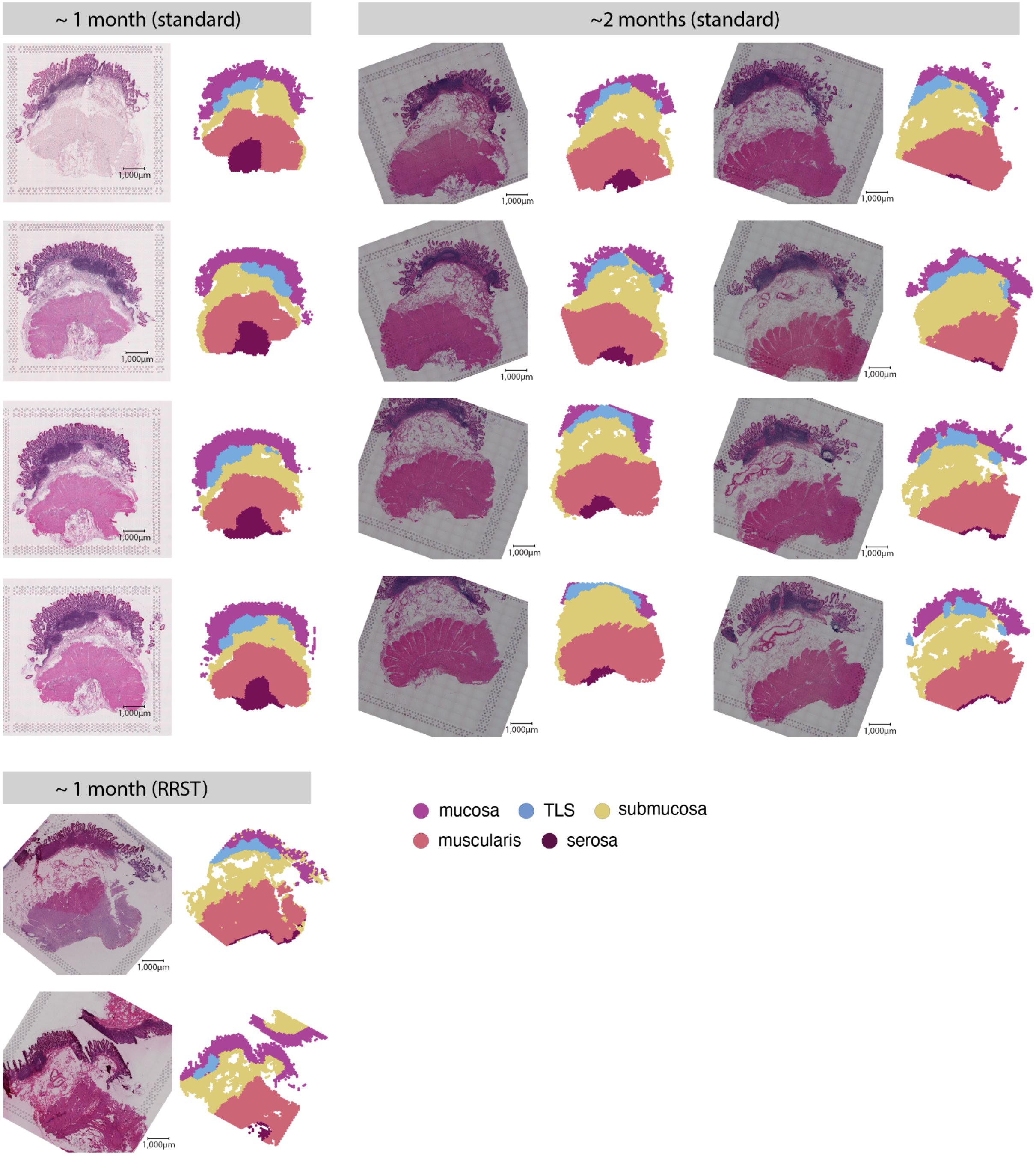

Supplementary Figure 9: Manual annotation of small intestine data. Each of the 14 spatial transcriptomics datasets were divided into 5 regions: mucosa (intestinal epithelium), submucosa, Tertiary Lymphoid Tissue (TLS), muscularis and serosa.

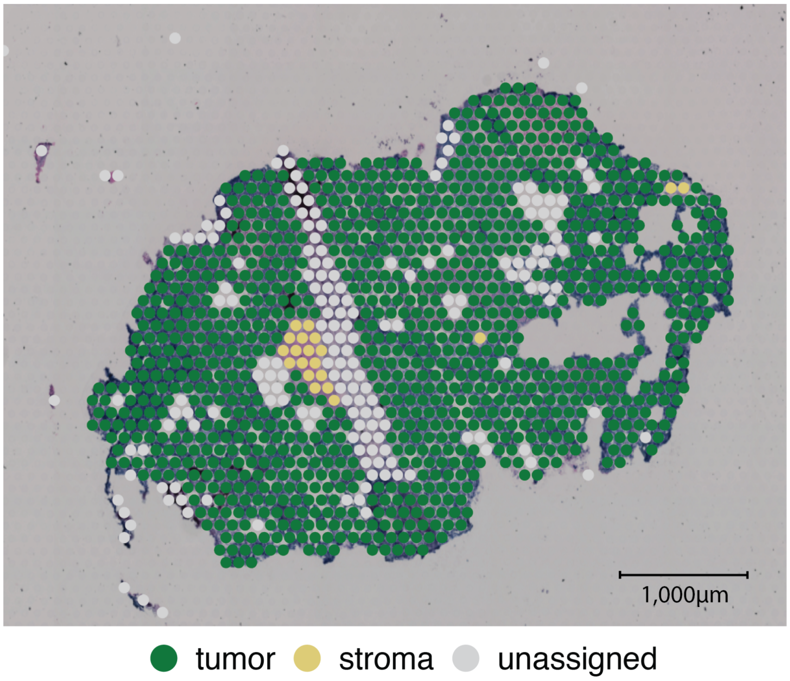

Supplementary Figure 10: Annotations made by a pathologist of a medulloblastoma tissue section.

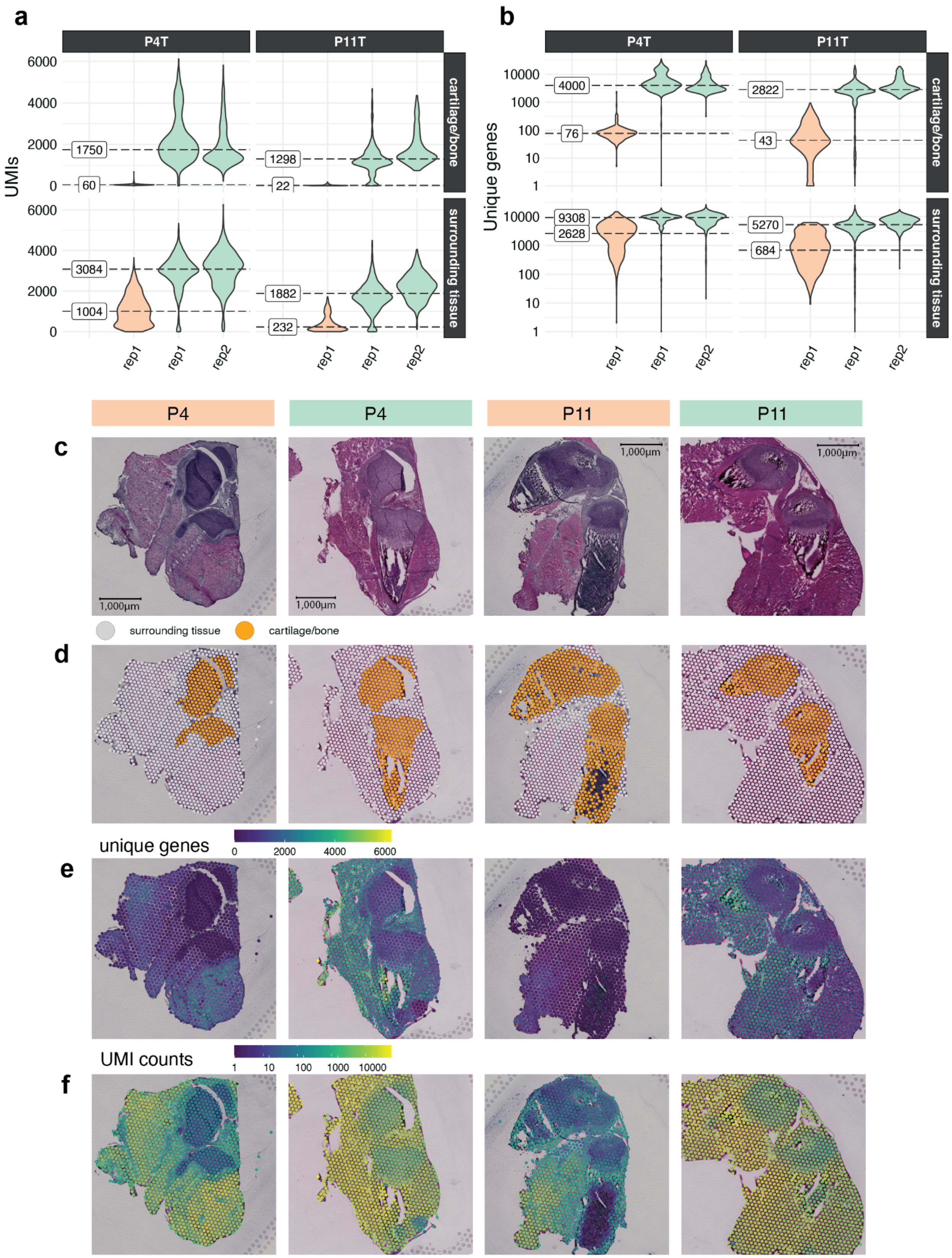

a
b
P4T
P11T
cartilage/bone
surrounding tissue
UMIs
Unique genes
1750
60
1298
22
3084
1004
1882
232
4000
76
2822
43
9308
2628
5270
684
rep1
rep2
P4
P11
c
d
e
f
1,000µm
surrounding tissue
cartilage/bone
unique genes
UMI counts

Supplementary Figure 11: Comparison between mouse bone data obtained with standard Visium and RRST. a) Distribution of unique genes per spot for all tissue sections visualized as violin plots. The plots are split by age (P4T or P11T) in columns and by tissue region (cartilage/bone or surrounding tissues) in rows. The median number of unique genes is highlighted on the left side of the violin plots for each tissue section, tissue region and protocol. **b)** Distribution of UMI counts per spot for all tissue sections visualized as violin plots with the y-axis converted into log10-scale. The plots are split by age (P4T or P11T) in columns and by tissue region (cartilage/bone or surrounding tissues) in rows. The median number of UMI counts is highlighted on the left side of the violin plots for each tissue section, tissue region and protocol. **c)** H&E images for 4 representative tissue sections, one for each age group and protocol. **d)** Spots colored by tissue region: cartilage/bone or surrounding tissue. **e)** Distribution of unique genes overlaid on H&E images. **e)** Distribution of UMI counts overlaid on H&E images. The colorbar represents log10-scaled UMI counts.

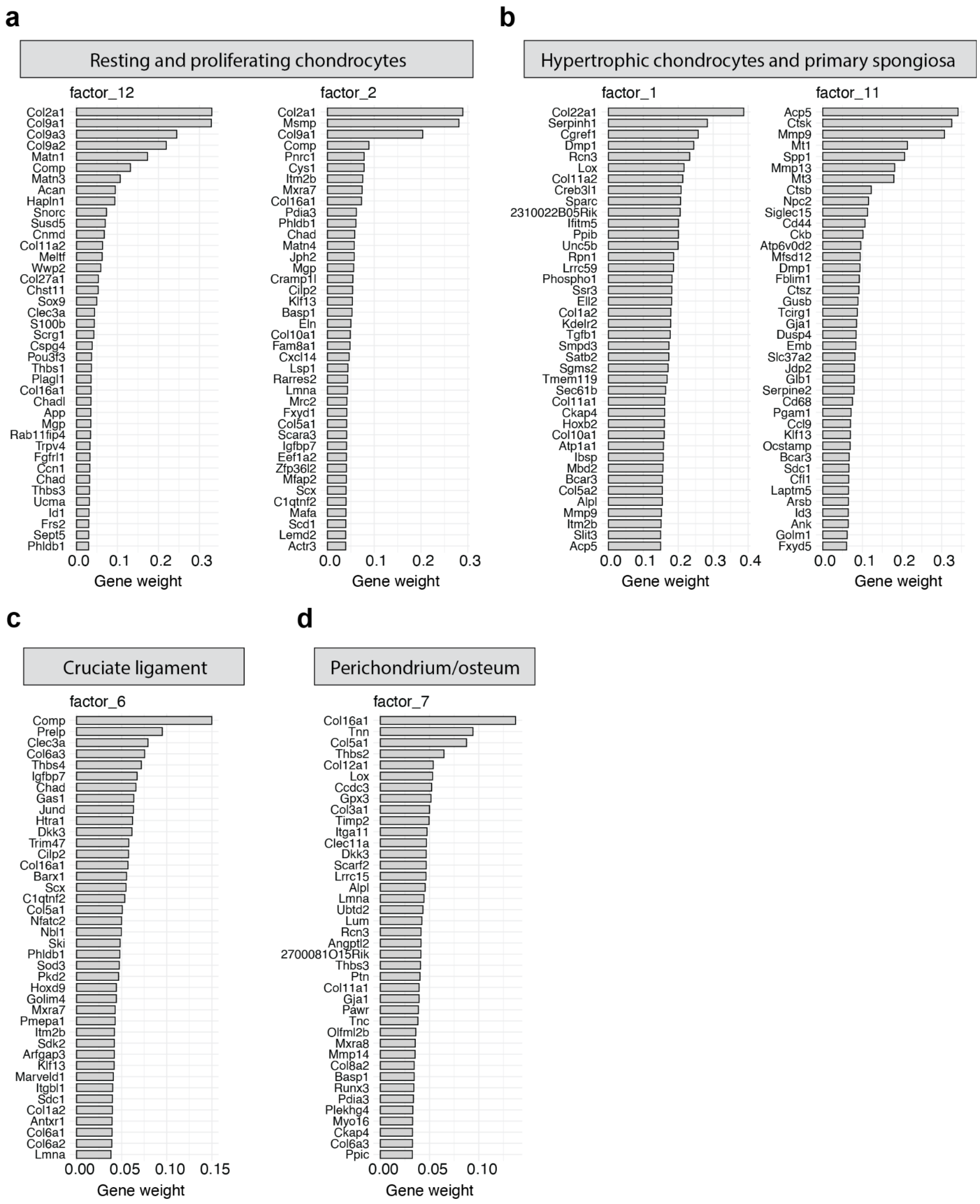

Supplementary Figure 12: NNMF analysis identified functions containing markers of resting and proliferating chondrocytes. (a), hypertrophic chondrocytes and cells within the primary spongiosa **(b)**, cruciate ligament **(c)** and cells at the perichondrium/periosteum **(d)**, which appeared in the expected, discrete anatomical locations (which are shown in **Fig. 6**).
