# Supplementary material for: Spatially resolved transcriptomic profiling of degraded and challenging fresh frozen samples
Source: Nat Commun. 2023 Jan 31;14:509. doi: 10.1038/s41467-023-36071-5 (PMC9889806; doi:10.1038/s41467-023-36071-5)
Supplement: Supplementary file 3 — Description of additional Supplementary File [file 41467_2023_36071_MOESM3_ESM.pdf]

Descriptions of additional supplementary files

Supplementary Data 1: Table with sample metadata

Supplementary Video 1: Tissue detachment
