# Supplementary material for: Spatially resolved transcriptomic profiling of degraded and challenging fresh frozen samples
Source: Nat Commun. 2023 Jan 31;14:509. doi: 10.1038/s41467-023-36071-5 (PMC9889806; doi:10.1038/s41467-023-36071-5)
Supplement: Supplementary file 6 — Reporting Summary [file 41467_2023_36071_MOESM6_ESM.pdf]

nature portfolio

Corresponding author(s): Joakim Lundeberg

Last updated by author(s): Dec 27, 2022

Reporting Summary

Nature Portfolio wishes to improve the reproducibility of the work that we publish. This form provides structure for consistency and transparency in reporting. For further information on Nature Portfolio policies, see our Editorial Policies and the Editorial Policy Checklist.

Statistics

For all statistical analyses, confirm that the following items are present in the figure legend, table legend, main text, or Methods section.

n/a	Confirmed	
☐	☒	The exact sample size (*n*) for each experimental group/condition, given as a discrete number and unit of measurement
☐	☒	A statement on whether measurements were taken from distinct samples or whether the same sample was measured repeatedly
☐	☒	The statistical test(s) used AND whether they are one- or two-sided<br>*Only common tests should be described solely by name; describe more complex techniques in the Methods section.*
☒	☐	A description of all covariates tested
☒	☐	A description of any assumptions or corrections, such as tests of normality and adjustment for multiple comparisons
☐	☒	A full description of the statistical parameters including central tendency (e.g. means) or other basic estimates (e.g. regression coefficient) AND variation (e.g. standard deviation) or associated estimates of uncertainty (e.g. confidence intervals)
☐	☒	For null hypothesis testing, the test statistic (e.g. *F*, *t*, *r*) with confidence intervals, effect sizes, degrees of freedom and *P* value noted<br>*Give P values as exact values whenever suitable.*
☒	☐	For Bayesian analysis, information on the choice of priors and Markov chain Monte Carlo settings
☒	☐	For hierarchical and complex designs, identification of the appropriate level for tests and full reporting of outcomes
☐	☒	Estimates of effect sizes (e.g. Cohen's *d*, Pearson's *r*), indicating how they were calculated

Our web collection on statistics for biologists contains articles on many of the points above.

Software and code

Policy information about availability of computer code

Data collection

All spatially resolved transcriptomics data generated in this study was processed from raw sequencing files in FASTQ format using the 10x Genomics Space Ranger softwarev1.2.1 or v1.3.1.

R distribution:
R v4.1.3

R packages:
uwot v0.1.11
ica v1.0-2
RcppEigen v0.3.3.9.2
Rcpp v1.0.8.3
cluster v2.1.3
sctranform v0.3.3
Seurat v4.1.0
ggbreak v0.0.9
ggpubr v0.4.0
magrittr v2.0.3
ggplot2 v3.3.5
ggrastr v1.0.1

openxlsx v4.2.5
viridis v0.6.2
hdf5r v1.3.5
scico v1.3.0
STUtility v1.1.1

web link to custom code:
https://github.com/ludvigla/RRST

Data analysis	All data analysis was carried out using the open source programming language R. Custom code used to run the analyses is available in our GitHub repository: https://github.com/ludvigla/RRST. A detailed specification of R package versions used for analyses can also be found in this repository. The code used to generate the figures as well as instructions for running the code inside a docker container are available at https://github.com/ludvigla/RRST.

For manuscripts utilizing custom algorithms or software that are central to the research but not yet described in published literature, software must be made available to editors and reviewers. We strongly encourage code deposition in a community repository (e.g. GitHub). See the Nature Portfolio guidelines for submitting code & software for further information.

Data

Policy information about availability of data

All manuscripts must include a data availability statement. This statement should provide the following information, where applicable:

- Accession codes, unique identifiers, or web links for publicly available datasets
- A description of any restrictions on data availability
- For clinical datasets or third party data, please ensure that the statement adheres to our policy

The following genomes used as references for data processing: GRCh38 v32 Ensembl 98 (Homo sapiens) and GRCm38 vM23 Ensembl 98 (Mus musculus). Both references were obtained from 10x Genomics website (reference: 2020-A).

All data required to replicate the analyses, including spaceranger output files, H&E images and additional files are available at Mendeley Data (DOIs for supplementary data: 10.17632/4w6krnywhn and 10.17632/442mhsrpcm.1).

Sequence data from the mouse brain and bone/cartilage samples have been deposited at GEO under accession number GSE221571. Sequence data for the pediatric brain tumors, colon/intestine, lung and prostate samples are available through a Materials Transfer Agreement with Monica Nister (monica.nister@ki.se), Guy Boeckxstaens (guy.boeckxstaens@kuleuven.be), Christos Samakovlis (Christos.Samakovlis@su.se) and Niklas Schultz (niklas.schultz@scilifelab.se), respectively in line with GDPR regulations. The data are available under Data Use 807 Conditions (DUO) and are limited to non-for-profit use as well as health/medical/biomedical 808 purposes. Access is granted if the above is fulfilled and local institutional review board/ethical 809 review board approvals are provided.

Human research participants

Policy information about studies involving human research participants and Sex and Gender in Research.

Reporting on sex and gender	Sex and gender are not considered and important for this study.
Population characteristics	Not relevant for this study.
Recruitment	No recruitment process was done in order to perform presented study. In this methodology paper, we aimed to demonstrate and benchmark our protocol of different tissue types. Individual samples were randomly provided from different ongoing projects. Patients operated for colorectal cancer or inflammatory bowel disease were recruited from a surgical ward with informed consent at University Hospitals Leuven. Lung tissues were obtained from deceased transplant organ donors by the Cambridge Biorepository for Translational Medicine (CBTM) with informed consent from the donor families and approval from the National Research Ethics Services (NRES) Committee of East of England. Patients who underwent radical prostatectomy were randomly selected by pathologist, written informed consent was obtained from all participants. Patients contributing with pediatric brain cancer samples were approached when operated for pediatric brain cancer.<br>There were no biases in the selection procedure for all samples.
Ethics oversight	Use of prostate cancer samples was approved by the Regional Ethical Review Board (REPN) Uppsala, Sweden before study initiation (Dnr 2011/066/2, Landstinget Västmanland, Sari Stenius).<br>Lung samples were obtained from deceased donors by the Cambridge Biorepository for Translational Medicine (CBTM) with informed consent from the donor families and approval from the NRES Committee of East of England – Cambridge South (15/EE/0152), the project has received funding from the European Union's Horizon 2020 research and innovation programme under a grant agreement (no. 874656, discovAIR).<br>GI tract specimens were approved by the medical ethics committee of University Hospitals Leuven (approval no. S62935).<br>Use of pediatric brain tumor samples was approved by the Regional Ethical Review Board (EPN), Stockholm, Sweden (DNR 2018/3-31, Monica Nister).

Note that full information on the approval of the study protocol must also be provided in the manuscript.

Field-specific reporting

Please select the one below that is the best fit for your research. If you are not sure, read the appropriate sections before making your selection.

- [x] Life sciences
- [ ] Behavioural & social sciences
- [ ] Ecological, evolutionary & environmental sciences

For a reference copy of the document with all sections, see nature.com/documents/nr-reporting-summary-flat.pdf

Life sciences study design

All studies must disclose on these points even when the disclosure is negative.

Sample size	No sample size calculation was preformed. Samples were selected based on RNA quality and previously failed experiments in order to demonstrate presented protocol. 52 tissue sections of different biological origins analyzed in this manuscript showing the versatility of the method, while some sample processed in replicates demonstrate the robustness. Given the high number of processed sections and variety of samples representing different tissue types we believe the sample size is sufficient to demonstrate the usability of presented method.
Data exclusions	No data were excluded from the analysis.
Replication	In order to verify the reproducibility of presented laboratory approach majority of the samples presented in this study were processed in technical replicates, where consecutive sections from the same tissue block were used to perform the experiments. In some cases biological replicates were used (sample from the same tissue from 2 different donors). In case of a mouse bone tissue, only one section per individual was used to perform standard Visium experiments due to the difficulty to obtain good quality data and the price for each experiment. All details can be found in Table 1 and Supplementary Data 1.
Randomization	Randomization of samples is not relevant for this study. In this work, samples were chosen based on RNA quality in order to perform comparison of 2 protocols, next samples were chosen based on biological origin to validate the method across different tissue types. As presented work is focused on method the randomization of samples/subjects is not of an importance in order to demonstrate its performance. Data analysis of all samples in this study was performed in groups according to the tissue origin (organ).
Blinding	Blinding was not relevant for this study. Samples from the same tissue specimens were analyzed together in order to make fair comparisons as each tissue is composed from different cell types that presents different challenges in sample processing and data generation.

Reporting for specific materials, systems and methods

We require information from authors about some types of materials, experimental systems and methods used in many studies. Here, indicate whether each material, system or method listed is relevant to your study. If you are not sure if a list item applies to your research, read the appropriate section before selecting a response.

Materials & experimental systems

n/a	Involved in the study	
x		Antibodies
x		Eukaryotic cell lines
x		Palaeontology and archaeology
	x	Animals and other organisms
x		Clinical data
x		Dual use research of concern

Methods

n/a	Involved in the study	
x		ChIP-seq
x		Flow cytometry
x		MRI-based neuroimaging

Animals and other research organisms

Policy information about studies involving animals; ARRIVE guidelines recommended for reporting animal research, and Sex and Gender in Research

Laboratory animals	Mouse brain samples were from C57BL/6J male mice of age 2 months and were commercially purchased from Adlego Biomedical. Mouse bone and cartilage samples were collected from postnatal mice (C57/BL6) at four and eleven days of age, sex of mice was not recorded due to difficulties to sex determination in this early stages of development. Mice were group-housed with the parent mouse on a 12h light-dark cycle, at 22°C with 50% humidity in the air.
Wild animals	Study did not involved wild animals.
Reporting on sex	Sex was not considered in this study. This work presents method for obtaining RNA sequencing data from tissue specimens of different biological origins and therefore sex is not important criterion.

Field-collected samples	This study did not involve field-collected samples.
Ethics oversight	Mouse bone samples were collected according to DNR 16673/2020, approved by Stockholm's animal experiment ethics committee (Stockholms djurförsöksetiska nämnd).<br>Mouse brain sample was purchased from Adlego Biomedical company, that operates under ethical permission nr. 17114-2020.

Note that full information on the approval of the study protocol must also be provided in the manuscript.
